# Multicenter Interlaboratory Study of Thrombin Generation Assay in Healthy Adults Using the ST Genesia Analyzer With the STG‐ThromboScreen Reagent (With and Without Thrombomodulin)

**DOI:** 10.1111/ijlh.70168

**Published:** 2026-06-09

**Authors:** Diego Velasco‐Rodríguez, María Marcos‐Jubilar, Inés Martínez‐Alfonzo, Laura Martos, Carlos Aguilar, Noelia Vilalta, Dolors Llobet, Ana Marco‐Rico, David Aragoneses, Pascual Marco, Luciana Ricca, David Ferreira, Sara Lopes, João Mariano‐Pego, Ángel Bernardo, Antonio Moscardó, Santiago Bonanad, Susana Gassiot, Isabel Gutiérrez‐Jomarrón, Jose Antonio Páramo, Pilar Llamas‐Sillero, Joan Carles Reverter

**Affiliations:** ^1^ Hospital Universitario Fundación Jiménez Díaz Madrid Spain; ^2^ Clínica Universidad de Navarra Pamplona Spain; ^3^ Diagnostica Stago S.A.S Asnières‐sur‐Seine France; ^4^ Hospital Santa Bárbara Soria Spain; ^5^ Hospital de la Santa Creu i Sant Pau Barcelona Spain; ^6^ Hospital General Universitario Doctor Balmis Alicante Spain; ^7^ Centro Hospitalar Universitário de São João Porto Portugal; ^8^ Centro Hospitalar Vila Nova de Gaia/Espinho Vila Nova de Gaia Portugal; ^9^ Centro Hospitalar Universitário de Coimbra Coimbra Portugal; ^10^ Hospital Universitario Central de Asturias Oviedo Spain; ^11^ Hospital Universitario La Fe Valencia Spain; ^12^ Hospital Sant Joan de Deu Barcelona Spain; ^13^ Hospital Universitario Príncipe de Asturias Alcalá de Henares Spain; ^14^ Hospital Clínic de Barcelona Barcelona Spain

**Keywords:** automated, interlaboratory, multicenter, thrombin generation

## Abstract

**Introduction:**

Fully automated thrombin generation (TG) assays facilitate the transition from research to clinical application. We conducted a multicenter inter‐laboratory study using the ST Genesia analyzer to evaluate assay performance and establish reference intervals in healthy adults from Spain and Portugal.

**Methods:**

In this multicenter study, plasma samples from healthy adult volunteers were analyzed across 12 laboratories following a harmonized protocol. TG was measured using the STG‐ThromboScreen reagent in the presence and absence of thrombomodulin (TM). Analytical performance and reference intervals were assessed.

**Results:**

Within‐run and intra‐day precision were excellent (coefficient of variation < 10%) for all TG parameters except velocity index. Normalization reduced interlaboratory variability for amplitude‐based parameters such as endogenous thrombin potential (ETP) and peak height, whereas its effect was less pronounced for velocity index. Satisfactory interlaboratory performance (|*z*| ≤ 2) was achieved in 96%–98% of results, and normalized parameters showed minimal bias across centers. In 252 healthy adults, median normalized values were: ETP 131% (IQR 118–142), peak height 116% (IQR 104–127), lag time 2.6 min (IQR 2.3–2.8), and time‐to‐peak 5.5 min (IQR 5.0–5.9). Median ETP inhibition in the presence of TM was 47.2% (IQR 35.1–61.8). Age had a modest influence on TG parameters, whereas sex differences were observed.

**Conclusions:**

This multicenter interlaboratory study in the Iberian Peninsula demonstrates high reproducibility of TG measurement using the ST Genesia platform with the STG‐ThromboScreen reagent. These findings support its use for standardized TG assessment and provide reference intervals for clinical and research settings.

## 1 Introduction

The thrombin generation (TG) assay is a global test that evaluates the entire coagulation process, unlike clotting time‐based assays, which only analyze the initial phases [1]. It explores thrombin formation, amplification, and inhibition in a continuous and simultaneous manner. Over the last decades, numerous studies have demonstrated that the TG assay (TGA) has the potential to assess both thrombotic and bleeding risk in many conditions [2].

However, the transition of TGA from research to routine has yet to be implemented. The calibrated automated thrombography (CAT), the most commonly used TGA in recent decades, lacks standardization and clinical validation. The reasons include differences in protocols between laboratories, preanalytical conditions and software versions, and different sources and concentrations of tissue factor (TF) and/or phospholipids [3, 4]. The development of ST Genesia (Diagnostica Stago), a fully‐automated analyzer, has overcome many of the above limitations. This method ensures a stable and uniform heat distribution throughout the measurements and allows normalization of TG parameters based on a reference plasma, which reduces the interlaboratory variation [5, 6].

Recently, several single‐center studies have been published in which normality ranges for TGA parameters have been defined using the ST Genesia analyzer in platelet‐poor plasma (PPP) samples of Caucasian healthy adults [7, 8, 9, 10, 11, 12]. Although their valuable contributions have laid the groundwork for the standardization of the TGA, their single‐center nature and lack of assessment of intrapersonal variability in most of them have been considered potential limitations.

Our objectives were (1) to evaluate the inter‐ and intra‐laboratory variability of ST Genesia with a multicenter approach, (2) to establish well‐defined normal reference intervals for all TGA parameters in the Spanish and Portuguese population, and (3) to demonstrate that TG assessment for research projects can be performed in a decentralized manner.

## 2 Methods

### 2.1 Study Design

This observational, prospective and multicentric study was conducted at nine Spanish [Hospital Universitario Fundación Jiménez Díaz (Madrid), Clínica Universidad de Navarra (Pamplona), Hospital de la Santa Creu i Sant Pau (Barcelona), Hospital Sant Joan de Deu (Barcelona), Hospital Universitario de la Fe (Valencia), Hospital Santa Bárbara (Soria), Hospital General Universitario Doctor Balmis (Alicante, Spain), Hospital Universitario Central de Asturias (Oviedo), Hospital Universitario Príncipe de Asturias (Alcalá de Henares)], and three Portuguese (Centro Hospitalar Universitário de São João, Centro Hospitalar Vila Nova de Gaia/Espinho, and Centro Hospitalar Universitário de Coimbra) centers. The investigators designed the study protocol and were responsible for data collection and analysis. The study followed the ethical principles of the Helsinki Declaration and was initially approved by the ethical committee of Hospital Universitario Fundación Jiménez Díaz in July 2022 and then by the ethical committee of all participating centers. Written informed consent was obtained from all volunteers.

The study comprised two phases:

- *Phase I:* “Precision, trueness and bias”
  Inclusion criteria were to be outpatients, older than 18 years and younger than 65 years and not having any significant disease, particularly no cardiovascular disease (except for arterial hypertension under treatment), including thrombotic episodes or bleeding disorders, cancer, liver disease, ongoing inflammatory conditions or any infectious disease. Exclusion criteria were the use of anticoagulants or any other drug interfering with coagulation such as oral contraceptives, hospitalization, previously known coagulation disorders, pregnancy and/or being younger than 18 years and older than 65 years.
  Three different pools were performed with at least 10 plasma donors and 3 aliquots of each one were sent to all the participants.
  Within‐run precision will refer specifically to the short‐term variability observed in three replicate measurements (R1, R2, R3) of the same aliquot performed under identical conditions within the same day and laboratory. Accuracy and bias were determined for the three pools separately with all the results obtained for each participant.
- *Phase II:* “The reference range”
  At least 25–30 healthy adult individuals for each participant center were included to define the reference range for each center. All individuals were selected according to CLSI guideline EP28‐A3 recommendation [13]. In phase II, demographic data (age and/or sex) were unavailable for 49 participants. These samples were excluded from the establishment of reference intervals, resulting in a final cohort of 252 individuals with complete demographic information.

### 2.2 Blood Collection and Processing

#### 2.2.1 Phase I: “Precision, Trueness and Bias”

In July 2022, three plasma pools were prepared using samples from 34 anonymized healthy adult volunteers at Hospital Universitario Fundación Jiménez Díaz. Blood was collected in 3.2% sodium citrate tubes (9:1 ratio) (Vacutainer, Becton Dickinson, Franklin Lakes, NJ, USA) via clean venipuncture with a 21 G needle. They were all de‐identified and anonymized after blood collection. There was neither a minimum number of patients for each pool, nor a minimum amount of plasma contributed by each patient, nor a fixed ratio of patients by age and sex.

Samples were free of hemolysis and lipemia. Platelet‐poor plasma (PPP) was obtained by double centrifugation (2500 g for 15 min at room temperature) and confirmed to contain platelet counts below 10 × 10^9^/L, being carefully handled to avoid foam generation. Plasmas from the same pool were combined, homogenized, and aliquoted (≥ 1.8 mL per tube) to obtain at least 45 aliquots per pool. Aliquots were stored at −80°C.

Frozen aliquots were shipped on dry ice to all participating centers under controlled conditions using validated couriers, with transport time not exceeding 24 h, and were frozen at −80°C until processing. Each working day, each participant thawed the samples in a 37°C water bath for exactly 10 min, following protocol. Phase I consisted of 3 working days at each site, with TG measured in triplicate each day using one aliquot from each pool.

#### 2.2.2 Phase II: “The Reference Range”

In this phase, 25–30 healthy individuals per center were included. One of the centers (Laboratory 5) did not participate in this phase because it is a pediatric hospital, and therefore its patients did not meet one of the inclusion criteria. All volunteers fulfilled the same inclusion and exclusion criteria applied in Phase I.

Two aliquots of PPP of at least 600 μL were collected for each individual. Blood collection, PPP collection, freezing, shipment and thawing were exactly as described for phase I. Samples were obtained between July and August 2022. TG measurement was carried out in 2 days; each day of analysis, 15 samples were processed.

Both demographic data [such as age, sex, and body mass index (BMI)] and TG parameters were recorded. The TG results of those healthy donors with missing demographic data or coagulation parameters were not taken into account for the Iberian reference range.

### 2.3 Thrombin Generation Assay

TG measurements were performed with the ST Genesia analyzer (Diagnostica Stago, Asnières‐sur‐Seine, France), using the STG‐ThromboScreen reagent in the presence or absence of thrombomodulin (TM) following the recommendations from the International Society of Thrombosis and Haemostasis (ISTH) [14]. The assay contained 3 quality controls (QC) (low, normal, and high) and reference plasma for parameter normalization. In the presence of both reagents, TG was triggered by dispensing a fluorogenic substrate and CaCl_2_. The STG‐ThromboScreen reagent contains recombinant human TF and phospholipids designed to trigger coagulation through the extrinsic pathway. The TF activity corresponds to a medium TF level comparable to the PPP reagent used in the CAT system. The exact TF concentration is proprietary and not publicly disclosed by the manufacturer. The kit includes paired reagents with and without thrombomodulin (TM). The TM‐containing reagent includes full‐length native thrombomodulin purified from rabbit lungs, calibrated to achieve approximately 50% inhibition of endogenous thrombin potential in normal plasma, allowing evaluation of the protein C anticoagulant pathway. Detailed information regarding the molecular structure or formulation of TM is not publicly disclosed by the manufacturer. For the entire study, the same batch of STG‐ThromboScreen reagents (LOT number: 202640) was used. The following parameters were measured: lag time, peak height, time‐to‐peak, ETP, ETP inhibition (%ETPinh), start tail and velocity index (vel.index). The STG‐ThromboScreen reagent was selected because it is currently the only reagent of the ST Genesia platform that is CE‐IVDR–marked for clinical use. Other reagents available for the system, such as STG‐BleedScreen and STG‐DrugScreen, are intended for research use only and have not been evaluated in the context of interlaboratory comparability studies. The use of a CE‐IVDR–marked reagent ensures compliance with regulatory requirements and supports the generation of clinically applicable and comparable results across laboratories.

### 2.4 Statistical Analysis

Data distribution was evaluated using the Shapiro–Wilk test. Continuous variables were expressed as median and interquartile range (IQR). For analytical performance evaluation, results were expressed as mean and standard deviation (SD). Qualitative variables were summarized using frequencies and percentages.

#### 2.4.1 Phase I: “Precision, Trueness and Bias”

For the within‐run precision analysis, the coefficient of variation (CV) of the three replicates (R1, R2, and R3) of each aliquot was calculated for all working days. Based on published literature [15], within‐run precision was considered acceptable when the CV of measurements from the same aliquot on the same day was < 10%.

For the within‐laboratory (inter‐day) analysis, once acceptable within‐run precision had been confirmed, the three replicate measurements from each day were averaged to obtain a single independent daily value per pool and per laboratory. These daily mean values were compared across laboratories using the non‐parametric Kruskal–Wallis test. When the global test was significant, pairwise comparisons were conducted using Dunn's post hoc test with Sidak correction for multiple comparisons.

To assess inter‐laboratory precision, the Precision Reproducibility Index (PRI) was calculated for each parameter (absolute and normalized values) as the ratio between the CV of a laboratory and the CV of the comparison group. The precision of a given laboratory was considered to be worse than that of the comparison group when the PRI value was greater than 1, whereas a PRI value of less than 1 suggested better precision than the comparison group. However, among the parameters with PRI greater than 1, we differentiated between those with PRI between 1 and 2 (acceptable, but the precision could be improved), and those with PRI greater than 2, which were flagged for review as potentially affected by analytical or preanalytical deviations, but their results were not excluded unless a documented technical issue was confirmed [16].

Trueness was assessed using *z*‐scores, calculated as the difference between the mean of each laboratory and that of the comparison group, divided by the standard deviation of the comparison group. Performance was considered satisfactory, questionable, or unsatisfactory when the absolute value of the *z*‐score of a parameter was ≤ 2; between 2 and 3; and ≥ 3, respectively, according to commonly accepted criteria in external quality assessment programs based on ISO 13528 [17, 18].

Bias was expressed as percentage relative error [15]:

$$\begin{array}{c} \text{Bias}\;\left(\%\right)=\left[100\times \left(\text{mean of laboratory}-\text{mean of comparison group}\right)\right]\\ \;/\text{mean of comparison group}. \end{array}$$

The relative error for each laboratory, pool, and parameter was considered acceptable when the bias was less than 10%.

#### 2.4.2 Phase II: “Reference Intervals”

For phase II, we first described values obtained from our population (median and interquartile range) as variables followed a non‐parametric distribution. According to CLSI document EP28‐A3c. 28—third edition 2008 and recent recommendations for coagulation testing [19], reference intervals were estimated as 2.5th percentile for the lower limit and 97.5th percentile for the upper limit [13]. As recommended by C28‐A3, we performed non‐parametric method of estimation [17, 18, 19, 20].

Associations between TG parameters and age were explored using linear regression. To evaluate the possible influence of age, the sample was divided into tertiles. In addition, differences by sex were analyzed, and women were divided into those older and those younger than 50 years to analyze the possible influence of estrogens [8]. Women using hormonal contraception were excluded from the reference population to avoid confounding effects on TG.

## 3 Results

### 3.1 Phase I

#### 3.1.1 Within‐Run Precision

The vast majority of CV values of the intra‐day replicate of each aliquot were less than 10% (Figure S1). However, not all parameters behaved equally. Both time‐dependent parameters (lag time, time‐to‐peak, and start tail) and ETP proved to show the highest within‐run precision. On the other hand, vel.index showed the highest variability, consistently exceeding 10% CV in most participating centers. During the study, a local technical issue related to reagent storage was identified in one participating laboratory. A temporary deviation from the recommended storage temperature occurred prior to analysis, affecting a limited number of runs performed during that specific period. These deviations were detected through internal QC procedures, including abnormal QC values and increased variability in replicate measurements. Only the results obtained during the affected runs were excluded from the inter‐laboratory analysis, whereas measurements performed before and after this event met predefined quality criteria and were therefore retained.

In addition, one laboratory showed slightly higher variability that was attributed to minor differences in pre‐analytical sample handling, particularly related to the timing of plasma thawing and preparation before analysis. These factors are known to influence TG kinetics and may contribute to increased variability in multicenter studies. Given the good within‐run precision observed, we took the averages of the repetitions of each pool performed each day to have a single value per pool per day. This is the value that was used to perform the within‐laboratory (inter‐day) precision analysis.

#### 3.1.2 Within‐Laboratory Precision

Some differences in the significance threshold were observed for some parameters between replicates performed on different days. In most laboratories, no pattern was observed for these differences in TG parameters or for any of the plasma pool samples. Nevertheless, differences between days were observed in one laboratory regarding ETP (*p* = 0.03) and in another laboratory regarding lag time (*p* = 0.04).

No laboratory (or ST Genesia analyzer) was considered to have unacceptable PRI‐based performance, nor did any of the TG parameters stand out from the rest by consistently presenting PRI > 2. Specifically, for pool 1, 100% of the participating centers had 4 or more of the 6 TG parameters for both absolute and normalized values with good or acceptable precision (PRI < 1 or PRI between 1–2, respectively). Only two laboratories, one for absolute values and one for normalized values, presented 2 of the 6 different TG parameters with a PRI > 2. For pool 2, 100% of the participants had 5 or more of the 6 TG parameters with a PRI < 2 for both absolute and normalized values. Only one laboratory and 3 laboratories for absolute and normalized values, respectively, had 1 of the 6 TG parameters with a PRI > 2. For pool 3, 100% had 4 or more of the 6 TG parameters with a PRI < 2 for both absolute and normalized values. Only two laboratories showed 2 of the 6 TG parameters with a PRI > 2 for normalized values, and one laboratory had only one TG parameter with a PRI > 2 for absolute values (Table S1).

Since adequate intra‐day precision was observed for each laboratory, we used the mean of each day's pool results in order to have a single value per laboratory for the purpose of inter‐laboratory comparisons.

#### 3.1.3 Inter‐Laboratory Analysis and Trueness

Although Dunn's test showed significant differences between some of the participants, when Sidak's correction was applied, it was observed that, in fact, there were no significant differences between the laboratory results for any of the pools (Figure S2).

Most parameters showed a CV < 10% for each pool, these differences being lower when normalized values were used (Table 1).

**TABLE 1:** Mean, standard deviation, and coefficient of variation of TG parameters: Inter‐laboratory assay (A: Absolute values, B: Normalized values).

A: Absolute values							
*N* = 12		Lag time (min)	Peak height (nM)	Time to peak (min)	ETP (nM.min)	Vel. index (nM/min)	Start tail (min)
Pool 1	Mean	2.2	221.8	5.2	1427.7	98.2	18.9
	SD	0.2	17.6	0.2	79.1	12.8	0.5
	CV (%)	3.2	7.9	3.6	5.5	13	2.8
Pool 2	Mean	2.3	209.3	5.2	1329.3	98.4	18.9
	SD	0.1	15	0.2	76.6	10.6	0.9
	CV (%)	3	7.2	3.7	5.8	10.8	4.7
Pool 3	Mean	2.2	211.8	5.2	1376.5	94.3	19.2
	SD	0.1	18.3	0.2	98	11.3	0.8
	CV (%)	2.8	8.6	3.6	7.1	12	4.1

B: Normalized values							
*N* = 12		Lag time (ratio)	Peak height (%)	Time to peak (ratio)	ETP (%)	Vel. index (%)	Start tail (ratio)
Pool 1	Mean	1.1	94.9	1.2	102	77.8	1
	SD	0.03	5.6	0.02	3.7	7.4	0.03
	CV (%)	2.8	5.9	1.9	3.6	9.4	2.6
Pool 2	Mean	1.1	91.5	1.2	96	80.7	1
	SD	0.03	5.3	0.03	3.1	7.4	0.04
	CV (%)	3.1	5.8	2.3	3.2	9.1	4.4
Pool 3	Mean	1.1	92.6	1.2	99.5	78.1	1
	SD	0.03	4.6	0.02	3.7	6.1	0.03
	CV (%)	2.8	5	1.9	3.7	7.8	2.9

*Note: N* = 12 refers to the number of participating laboratories included in the analytical evaluation. Three plasma pools were prepared from platelet‐poor plasma obtained from healthy adult volunteers without known coagulation disorders or anticoagulant treatment. The pools were designed to represent normal coagulation conditions and were used for the assessment of analytical precision and reproducibility across laboratories.

A satisfactory performance (*z*‐score ≤ 2) was observed in 96.2% of TG parameters for pools 1 and 2, and 98% for pool 3. No participating center presented unsatisfactory performance (*z*‐score ≥ 3) in any parameter in any of the three pools. Detailed *z*‐score values for each parameter and laboratory are provided in Table 2.

**TABLE 2:** Trueness analysis.

Pool	Lab ID	Lag time (min)	Peak height (nM)	Time to peak (min)	ETP (nM.min)	Vel. index (nM/min)	Start tail (min)	Lag time (ratio)	Peak height (%)	Time to peak (ratio)	ETP (%)	Vel. index (%)	Start tail (ratio)	ETP% Inh
1	1	0.96	0.02	0.41	0.37	−0.06	0.94	1.14	−0.69	0.79	−0.64	−0.90	0.64	−1.40
	2	−1.25	−0.13	−0.51	−0.34	−0.15	−0.63	−0.33	−1.59	0.56	−1.81	−1.15	0.29	1.01
	3	0.48	−0.82	0.18	−0.79	−0.49	0.95	0.81	0.51	0.17	1.26	0.30	0.95	0.07
	4	0.12	1.87	−0.60	**2.15**	1.34	−0.62	−0.11	1.66	−1.33	1.55	1.43	−1.14	0.89
	5	0.18	−1.14	1.66	−0.67	−1.44	1.21	−1.26	0.13	0.71	0.63	−0.43	−0.08	**−2.08**
	6	0.48	0.45	−0.78	0.61	0.88	0.38	−0.48	0.41	−0.21	0.63	0.41	0.29	−0.07
	7	0.01	−0.06	0.35	0.09	−0.25	−0.03	−0.61	−0.38	0.20	−0.30	−0.61	0.12	−0.25
	5	−1.22	0.43	0.07	0.64	−0.22	−0.80	**−2.03**	0.08	−0.58	−0.32	−0.12	−1.27	1.01
	9	0.20	1.77	−1.46	0.95	**2.12**	−1.67	0.72	1.34	**−2.40**	−0.26	2.22	−1.69	−0.73
	10	−0.87	−0.95	−0.13	−1.16	−0.46	0.29	0.12	−0.82	0.45	0.00	−0.60	1.64	0.59
	11	**2.08**	−0.90	1.86	−0.53	−1.13	1.27	1.02	0.66	0.67	0.63	0.26	−0.59	0.97
	12	−1.18	−0.55	−1.05	−1.32	−0.13	−1.30	1.02	−1.32	0.97	−1.38	−0.80	0.84	−0.03
2	1	0.93	−0.65	1.10	0.50	−0.92	1.71	0.83	−1.76	1.86	−0.64	−1.94	1.64	−0.45
	2	−0.71	0.05	−0.69	0.06	0.43	−0.80	0.35	−1.17	0.31	−1.53	−0.88	0.22	0.91
	3	0.05	−0.96	0.09	−0.76	−0.69	0.62	0.18	0.19	0.09	1.21	−0.01	0.63	0.42
	4	0.57	1.78	−0.11	**2.16**	1.19	0.24	0.32	1.12	−0.53	1.70	0.99	−0.07	0.18
	5	−0.74	−0.15	0.19	−1.14	−0.43	−1.50	−1.52	1.42	−0.57	−0.43	0.65	**−2.21**	0.66
	6	0.15	−1.06	1.04	−0.64	−1.31	0.34	−1.16	0.31	−0.04	0.52	−0.15	−0.47	−1.27
	7	0.56	0.31	0.05	0.56	0.26	0.40	0.00	−0.16	−0.20	0.34	−0.28	0.52	0.00
	5	−1.16	0.54	0.04	0.70	−0.14	−0.27	−1.59	−0.08	−0.54	−0.37	−0.19	−0.57	1.05
	9	−0.31	1.89	−1.27	0.83	**2.15**	−1.13	0.13	1.13	−1.97	−0.61	**2.05**	−1.10	0.11
	10	−1.22	−0.33	−1.47	−1.16	0.44	−0.64	−0.23	−0.12	−0.54	−0.27	0.17	0.14	**−2.09**
	11	**2.31**	−1.06	1.91	−0.14	−1.11	1.55	0.97	0.40	0.86	1.21	0.34	0.39	1.27
	12	−0.42	−0.37	−0.87	−0.96	0.13	−0.52	1.70	−1.28	1.25	−1.13	−0.75	0.88	−0.79
3	1	1.25	−0.34	0.60	0.26	−0.36	1.35	1.18	−1.58	1.51	−0.83	−1.70	1.55	−1.33
	2	−1.34	0.88	−1.23	0.22	1.05	−1.35	−0.24	−0.36	−0.62	−1.02	−0.16	−0.68	−0.27
	3	0.32	−1.35	0.48	−0.98	−1.04	1.21	0.47	−0.84	0.88	0.21	−0.67	1.50	0.84
	4	0.22	1.78	−0.15	**2.34**	1.29	0.30	−0.06	1.97	−0.76	**2.80**	1.59	0.01	0.73
	5	−0.12	−0.63	0.48	−0.87	−0.80	−0.55	−0.99	0.65	−0.30	−0.28	0.09	−1.43	0.41
	6	0.56	−1.12	0.96	−0.72	−1.17	0.90	−0.84	−0.19	−0.27	−0.06	−0.20	−0.05	−0.62
	7	0.45	−0.12	0.13	0.23	−0.10	0.65	−0.16	−0.72	0.12	−0.09	−0.73	1.05	0.00
	5	−1.27	0.80	0.04	0.94	0.10	−0.51	−1.90	0.65	−0.72	0.47	0.14	−0.99	1.29
	9	−0.91	1.32	−0.71	0.78	1.47	−0.97	−0.45	1.04	−1.52	−0.30	1.87	−1.12	1.36
	10	−0.53	−0.04	−1.54	−0.85	0.80	−1.06	0.63	0.32	−0.86	−0.04	0.75	−0.19	−1.93
	11	1.97	−1.02	1.95	−0.58	−1.46	0.99	0.63	0.15	1.23	0.13	−0.41	−0.29	−0.12
	12	−0.61	−0.17	−1.01	−0.77	0.23	−0.98	1.73	−1.09	1.32	−1.00	−0.56	0.64	−0.35

*Note:* The performance of each laboratory was considered satisfactory, questionable, or unsatisfactory when the absolute value of the *z*‐score of a parameter was ≤ 2; between 2 and 3; and ≥ 3, respectively. Bold values represent absolute values of *z*‐score considered non‐satisfactory.

#### 3.1.4 Bias

Finally, the majority of centers showed an acceptable relative error for most TG parameters (Table S2). However, a systematic error in peak height for laboratories 4 and 9 and in ETP for laboratory 4 was observed in all three pools, but only in absolute values. Again, vel.index was the parameter that stood out negatively, with bias often greater than 10%. Except for vel.index, no systematic error was detected in the normalized TG parameters for any pool or laboratory.

### 3.2 Phase II

Information on age and sex was not available for 49 patients, thus they were excluded from the analysis. A total of 252 individuals were finally included, with a median age of 41 years old (18–65), of which 61.3% were female. The median BMI was 24.5 kg/m^2^ (18.5–33.6). There were significant differences in age, sex, and BMI between laboratories.

The association between age and TG parameters was evaluated, and it was observed that, except for ETP, there was a significant association. In addition, the TG parameters were influenced by sex, except for lag time and start tail. Thus, we evaluated the differences in TG parameters between laboratories, adjusting for age and sex, showing that except for ETP inhibition, there were no differences in TG parameters between laboratories. Thus, we established normal values ranges for all TG parameters depending on age and sex (Table 3).

**TABLE 3:** Age‐ and sex‐stratified reference intervals for thrombin generation parameters in healthy adult subjects.

	< 50 years		> 50 years	
	Female (*n* = 113)	Male (*n* = 62)	Female (*n* = 41)	Male (*n* = 35)
	*p*2.5–97.5	*p*2.5–97.5	*p*2.5–97.5	*p*2.5–97.5
Normalized data (*n* = 251)				
Lag time (ratio)	0.8–1.4	0.7–1.5	0.8–1.8	0.7–1.7
Peak height (%)	47–145	58–112	47–140	54–135
Time to peak (ratio)	0.7–1.5	0.8–1.6	0.8–1.6	0.8–1.6
ETP (%)	69–126	64–120	69–127	71–124
Velocity index (%)	31–133	45–92	17–121	33–113
Start tail (ratio)	0.7–1.4	0.7–1.3	0.8–1.4	0.7–1.3
ETP inh (%)	22–59	36–88	20–84	43–76

*Note:* Age stratification was performed because several TG parameters showed a significant association with age in the regression analysis, and age‐related changes in coagulation factors are known to influence thrombin generation. Bold values represent absolute values of *z*‐score considered non‐satisfactory.

Abbreviation: *p*, percentile.

## 4 Discussion

We conducted the first multicenter interlaboratory study of fully‐automated TG using plasma samples from healthy adult donors under standardized preanalytical conditions. This study provides real‐world data across the Iberian Peninsula and externally validates the ST Genesia platform with the STG‐ThromboScreen reagent. The present study shows that processing plasmas with the same working protocol using the fully automated ST Genesia analyzer and the STG‐ThromboScreen reagent resulted in excellent precision and accuracy across independent laboratories, all of which are key concepts in laboratory quality [21, 22]. Plasma used for analytical evaluation was obtained from healthy adult volunteers without relevant medical conditions or medication known to interfere with coagulation. This ensured that pooled samples were representative of normal coagulation profiles and minimized biological variability in the assessment of analytical performance.

A small proportion of intraday CV values > 10% highlights the importance of standardized preanalytical conditions, particularly minimizing the time between plasma thawing and analysis (< 1 h). Increased variability in peak height under low tissue factor conditions has been previously reported, underscoring the need for standardization [23]. We obtained an adequate within‐laboratory precision, with no pattern observed despite having some differences for some parameters between working days. Most parameters showed a PRI < 1, indicating very good intra‐laboratory reproducibility. Conversely, laboratories with a high PRI, particularly those with values > 2, may reflect pre‐analytical issues or inconsistencies in test procedures.

Inter‐laboratory results across ST‐Genesia analyzers were consistent, with repeatability CV < 5% and reproducibility CV < 10% for most parameters; after normalization, inter‐laboratory reproducibility fell below 6%, and mean biases versus assigned QC values were < 5% [24].

Vel.index consistently showed higher intra‐day and inter‐laboratory CV values. As a derived parameter calculated from peak height, time‐to‐peak, and lag time [25], small variations in primary measurements are amplified. Similar findings have been reported by van Paridon et al. [9] and Dargaud et al. [23]. Occasional variability in peak height likely reflects its higher analytical sensitivity to pre‐analytical and instrumental differences. Despite this, overall reproducibility remained within acceptable limits. One of the advantages of ST Genesia with respect to semi‐automated methods such as CAT (Diagnostica Stago) is the normalization of the parameters with reference plasma. As reported in other publications and in other assays, normalization leads to better reproducibility, reduces inter‐assay and inter‐laboratory variation, and allows comparison between laboratories and facilitates the interpretation of the results [10, 11, 26]. Normalization reduced inter‐laboratory variability, particularly for amplitude‐based parameters such as ETP and peak height. However, its effect was less pronounced for velocity index, likely due to its composite nature, as it is derived from multiple TG parameters. As a result, normalization corrects systematic analytical differences but cannot fully compensate for variability inherent to derived kinetic variables. Similar observations have been reported in previous studies.

Additionally, ST Genesia has been CE‐IVDR certified for clinical use, presents better traceability, and its reagents are pre‐dosed in ready‐to‐use cartridges and include an integrated QC system [27]. Long‐term analytical evaluations have further confirmed the stability and reproducibility of ST Genesia performance over extended periods of routine use [28]. In contrast, the CAT system relies on an internal calibration curve using a thrombin calibrator added to each plasma sample, whereas ST Genesia applies an external calibration based on a reference plasma analyzed separately and subsequently used to normalize the results. This fundamental difference in calibration strategy contributes to improved between‐run and inter‐laboratory reproducibility with ST Genesia, but also explains systematic discrepancies in reported TG parameters between platforms. Several comparative studies have shown that CAT typically yields higher absolute ETP and peak height than ST Genesia, whereas lag time and time‐to‐peak tend to be shorter with CAT [10, 29, 30]. These differences are mainly attributed to distinct calibration algorithms, signal processing, and reagent formulations, underscoring that results obtained with both systems are not directly interchangeable [28, 29, 31, 32]. However, both methods consistently reflect the overall TG potential of the sample, and within‐system normalization allows reliable intra‐ and inter‐laboratory comparison.

A similar two‐center interlaboratory comparability study using the ST Genesia method has been recently published, reporting satisfactory precision, accuracy, and inter‐laboratory variability using QC and plasma samples from both healthy adult volunteers and patients [24]. However, it has several limitations. First of all, the impact of preanalytical conditions on the assay was not assessed. Moreover, neither vel.index (whose CV is known to be higher) nor start tail were analyzed. Start tail corresponds to the time point at which TG returns to baseline levels and therefore reflects the termination phase of thrombin generation, which is influenced by endogenous anticoagulant mechanisms responsible for thrombin neutralization. Although less frequently reported than other TG parameters, it may provide complementary information on the global coagulation profile.

Our results suggest that TG can be reliably measured in a decentralized manner. Overall, the participating laboratories achieved consistent results after normalization, although one site exhibited higher variability attributed to pre‐analytical factors. Closer inspection of individual laboratory performance revealed that laboratories 3 and 4 showed slightly higher variability for certain parameters, particularly normalized ETP. Interestingly, laboratory 3 showed values aligned with other centers when absolute parameters were considered, whereas the deviation appeared after normalization. This finding likely reflects minor variations in the measurement of the reference plasma used for normalization, since normalized results depend on the ratio between the sample and reference plasma values. Small analytical differences in reference plasma measurements may therefore propagate into normalized parameters without necessarily affecting absolute values. Such observations highlight the importance of strict adherence to standardized analytical procedures and careful QC when implementing normalization strategies in multicenter settings.

This innovative approach may facilitate the development of collaborative laboratory networks, improving access to TG testing and enabling multicenter clinical studies. In the second phase of the study, we determined the reference ranges of the ST Genesia analyzer for STG‐ThromboScreen in a healthy adult population. Since our study included multiple centers across Spain and Portugal, the resulting reference values can be considered representative of the south‐western European population. To date, five single‐center studies on TG reference ranges based on the ST Genesia method have been published [7, 8, 10, 33, 34]. Almost all of them have used the STG‐ThromboScreen reagent, and two of them also used the STG‐DrugScreen reagent [7, 34]. Whereas the studies by Ninivaggi et al., Calzavarini et, Carlo et al. and Kristensen et al. established reference ranges in adult individuals, other works have focused on ranges in the pediatric population [33, 34]. Regarding the TG technology, Omasombo et al. also found statistically significant differences between the ST Genesia method and CAT in both peak height and ETP [34].

Our reference intervals were obtained by combining data from relatively small but well‐characterized cohorts across multiple centers, following CLSI EP28‐A3c recommendations for multicenter studies. This approach allowed us to capture biological variability under real‐world laboratory conditions while ensuring preanalytical harmonization. Despite differences in sample size and demographics, our results are consistent with those reported in previous studies using the same automated platform. For instance, Dargaud et al. [23] and Van Paridon et al. [9] reported comparable ETP and peak height values in healthy adults from France and the Netherlands, respectively. Likewise, Biss et al. [35] confirmed similar reference intervals and reproducibility metrics for ST Genesia. These concordant findings reinforce the reliability of our data and support the analytical robustness of the STG‐ThromboScreen reagent across different European populations. A summary of published reference intervals obtained with the ST Genesia platform in healthy adults is provided in Table S3.

Sex‐ and age‐related differences in TG parameters were also evident. Men showed higher values than women under 50 years, whereas women exceeded men after menopause. Similar trends were reported by Castoldi et al. [36], whereas Van Paridon et al. [9] found attenuated sex differences using the same automated platform. These findings highlight the hormonal modulation of TG and the need for sex‐ and age‐specific reference intervals. The differences observed in %ETP inhibition across age and sex strata may also reflect physiological variability in the sensitivity of thrombin generation to the protein C anticoagulant pathway, which is known to be influenced by hormonal status and age‐related changes in coagulation factors. Unlike most previously published studies, which were based on smaller sample sizes [7, 8, 10, 11], and often failed to stratify results by age and sex [8], our study provides a more granular and reliable description of physiological variability. Additionally, while other studies included individuals up to 80 years of age, we excluded individuals over 65, ensuring a more homogeneous and healthier reference population with lower expected factor VIII levels—an important determinant of TG [37]. A further strength of our study is the exclusion of women under hormonal influence, including both young women taking hormonal contraceptives and older women undergoing hormone replacement therapy [38]. This rigorous exclusion criterion contributes to the robustness and interpretability of our results and enhances the validity of the reference ranges we propose.

Regarding population differences, we acknowledge that TG parameters may vary among geographic and ethnic groups due to genetic, dietary, and environmental factors influencing hemostatic balance. Nevertheless, studies performed with ST Genesia in other Western European cohorts, including French and Dutch populations, have reported comparable TG ranges and distributions [9, 23]. These findings support the analytical consistency of the system across populations with similar demographic and lifestyle characteristics. Therefore, although minor inter‐population differences cannot be completely excluded, our results align with previously published European data and provide robust, locally validated reference intervals for the Iberian population.

The Iberian range will serve as a reference for all participating centers, to detect whether their TG assessment is working well or whether any aspect of their work protocol, such as the pre‐analytical phase or patient selection, needs to be revised. However, as with any other laboratory technique, it is advisable for each center to calculate its own normality ranges. This study has several strengths, including its multicenter design, standardized preanalytical protocol, and the large number of healthy adult participants. In addition to establishing reference intervals, it provides robust evidence that TG measurements are reproducible across laboratories using a harmonized methodology.

On the other hand, it has also some possible limitations. Phase I included a relatively limited number of participating centers (*n* = 12) and repetitions, although the robustness of the results mitigates this constraint. In addition, the study was restricted to PPP and to the STG‐ThromboScreen reagent, with and without TM. This choice was intentional, as STG‐ThromboScreen is currently the only CE‐IVDR–marked and fully standardized reagent validated for clinical use on the ST Genesia platform. Restricting the analysis to this reagent ensured methodological consistency, regulatory compliance, and reliable inter‐laboratory comparison. Future studies including other ST Genesia reagents or additional matrices such as platelet‐rich plasma may further expand the analytical scope and clinical applicability of the system. Another limitation of phase II is that only Caucasian individuals were included. While this homogeneity enhances internal consistency and minimizes genetic and environmental variability, it limits the generalizability of the reference ranges to other ethnic groups. Future studies including more diverse populations would be valuable to confirm the applicability of these reference intervals across different demographic backgrounds. Moreover, the same batch of reagents and calibrators was used for both study phases. This approach minimized analytical variability and ensured consistency across all measurements. However, as a result, potential lot‐to‐lot variability could not be assessed, which should be addressed in future inter‐laboratory evaluations. Finally, our findings do not apply to STG‐BleedScreen and STG‐DrugScreen reagents, as they have not been evaluated in this study.

The relatively wide reference intervals observed in our study reflect the inherent interindividual variability of TG, which integrates multiple physiological determinants of hemostasis, as well as the inclusion of data from multiple centers. Although this multicenter approach may increase dispersion, it provides more representative and generalizable reference limits for the Iberian population. Similar variability has been reported in previous studies using the ST Genesia platform (Table S3), underscoring the influence of demographic, pre‐analytical, and analytical factors on TG parameters. In clinical practice, abnormal TG profiles should be interpreted relative to both the population reference intervals and the internal laboratory controls, considering deviations in normalized parameters—particularly ETP and peak height—as potentially indicative of hyper‐ or hypocoagulable states. Standardization of interpretation criteria, supported by outcome‐based studies, will be essential to define clinical decision thresholds in the future.

In summary, TG measurement using the ST Genesia analyzer with the STG‐ThromboScreen showed robust analytical performance in healthy adult subjects. The reference intervals established in this multicenter study may serve as a useful framework for future clinical investigations using this methodology. The implementation of the ST Genesia system with standardized procedures may facilitate broader clinical application of TG testing in hemostasis research and laboratory practice.

## Author Contributions

D.V.‐R., M.M.‐J., L.M., I.M.‐A., J.A.P, P.L.‐S. and J.C.R. conceived and designed the study. All authors contributed to data acquisition. M.M.‐J. performed the statistical analysis. D.V.‐R. drafted the manuscript. All authors critically revised the manuscript and approved the final version.

## Funding

This work was supported by the Diagnostica Stago. The company provided study reagents and technical support.

## Ethics Statement

The study was conducted in accordance with the Declaration of Helsinki and was approved by the Ethics Committee of Hospital Universitario Fundación Jiménez Díaz and by the corresponding ethics committees of all participating centers.

## Consent

Written informed consent was obtained from all participants prior to inclusion in the study.

## Conflicts of Interest

L.M. is an employee of Diagnostica Stago. The remaining authors declare no conflicts of interest.

## Supporting information


**Figure S1:** Coefficients of Variation (CV) for intraday replicates of each plasma aliquot. Each cell represents the CV for a single aliquot. Green cells indicate CV value below 10%, while red cells indicate CV value above 10%. Each CV value represents the mean of three intraday replicates (R1, R2, and R3).

**Figure S2:** Inter‐laboratory comparison of daily thrombin generation results. For each laboratory and plasma pool, three intraday replicate measurements (R1, R2, and R3) were obtained from the same aliquot and averaged to obtain a single daily value. Daily values are displayed for each laboratory. Panel A shows absolute values and Panel B normalized values. Between‐laboratory comparisons were performed using the Kruskal–Wallis test followed by Dunn's post hoc test with Sidak correction for multiple comparisons.

**Table S1:** Within‐laboratory analysis, expressed by the Precision Reproducibility Index (PRI) of each parameter in all laboratories. PRI was calculated as the ratio between the CV of a laboratory and the CV of the comparison group (PRI = CVlab/CVgroup). PRI < 1 indicates better precision than the comparison group, whereas PRI > 1 indicates worse precision. Values between 1 and 2 were considered acceptable, while PRI > 2 indicated poor reproducibility and prompted internal review. The table shows how many of the 6 TG parameters evaluated in the study for absolute and normalized values presented a PRI < 1, between 1–2 or > 2.

**Table S2:** Bias analysis. The relative error for each laboratory, pool and parameter was considered acceptable when the bias was less than 10%.

**Table S3A:** Published reference intervals for thrombin generation parameters (absolute values) on ST Genesia using STG‐ThromboScreen.
**Table S3B:** Published reference intervals for normalized thrombin generation parameters and %ETP inhibition on ST Genesia using STG‐ThromboScreen.

## Data Availability

The data that support the findings of this study are available on request from the corresponding author. The data are not publicly available due to privacy or ethical restrictions.
