# Supplementary material for: Multicenter Interlaboratory Study of Thrombin Generation Assay in Healthy Adults Using the ST Genesia Analyzer With the STG‐ThromboScreen Reagent (With and Without Thrombomodulin)
Source: Int J Lab Hematol. 2026 Jun 9;48(5):1189–200. doi: 10.1111/ijlh.70168 (PMC13555124; doi:10.1111/ijlh.70168)
Supplement: Supplementary file 1 [file IJLH-48-1189-s001.docx]

**Supplementary Figure S1.** **Coefficients of Variation (CV) for intraday replicates of each plasma aliquot.**  Each cell represents the CV for a single aliquot. Green cells indicate CV value below 10%, while red cells indicate CV value above 10%. Each CV value represents the mean of three intraday replicates (R1, R2 and R3).
