# Supplementary material for: Multicenter Interlaboratory Study of Thrombin Generation Assay in Healthy Adults Using the ST Genesia Analyzer With the STG‐ThromboScreen Reagent (With and Without Thrombomodulin)
Source: Int J Lab Hematol. 2026 Jun 9;48(5):1189–200. doi: 10.1111/ijlh.70168 (PMC13555124; doi:10.1111/ijlh.70168)
Supplement: Supplementary file 2 [file IJLH-48-1189-s002.docx]

**Supplementary Table S3A. Published reference intervals for thrombin generation parameters (absolute values) on ST Genesia^®^ using STG-ThromboScreen^®^**

| **Study** | **N** | **Lag time (min)** | **Time to peak (min)** | **Peak height (nM)** | **ETP (nM·min)** | **Velocity index (nM/min)** | **Start tail (min)** |
| --- | --- | --- | --- | --- | --- | --- | --- |
| Calzavarini et al., 2019 | 123 | NR | NR | NR | NR | NR | NR |
| Ninivaggi et al., 2020 | 117 | 2.1–2.7 | 4.4–6.6 | 180–266 | 1028–1303 | 41.8–154.2 | 14.2–20.0 |
| Kristensen et al., 2022 | 40 | 1.7–2.9 | 4.3–6.5 | 197–361 | 1336–1973 | 53–131 | NR |

**Abbreviations:** NR, not reported.
**Notes:** Kristensen et al. reported median [2.5–97.5 percentiles] and included women using combined hormonal contraception; values shown correspond to the −TM condition. Only studies performed using STG-ThromboScreen^®^ on platelet-poor plasma were included.

**Supplementary Table S3B. Published reference intervals for normalized thrombin generation parameters and %ETP inhibition on ST Genesia^®^ using STG-ThromboScreen^®^**

| **Study** | **N** | **Normalized lag time (ratio)** | **Normalized time to peak (ratio)** | **Normalized peak height (%)** | **Normalized ETP (%)** | **Normalized velocity index (%)** | **Normalized start tail (ratio)** | **%ETPinh** |
| --- | --- | --- | --- | --- | --- | --- | --- | --- |
| Calzavarini et al., 2019 | 123 | 1.0–1.3 | 1.0–1.3 | 45–102 | 59–99 | 60–73 | NR | 49–80 |
| Ninivaggi et al., 2020 | 117 | 0.9–1.2 | 0.9–1.5 | 60–90 | 66–105 | 32–110 | 0.8–1.2 | 30–66 |
| Kristensen et al., 2022 | 40 | NR | NR | NR | NR | NR | NR | 22–40 |

**Abbreviations:** %ETPinh, percentage of ETP inhibition in presence of thrombomodulin; NR, not reported.

**Notes:** Calzavarini et al. reported only normalized ratios vs reference plasma. Ninivaggi et al. reported both absolute and normalized parameters using STG-ThromboScreen®. Kristensen et al. reported %ETP inhibition (TM-condition) as median [2.5–97.5 percentiles] but did not report normalized values. Only studies performed on platelet-poor plasma using the STG-ThromboScreen^®^ reagent were included.
