# Supplementary material for: Multicenter Interlaboratory Study of Thrombin Generation Assay in Healthy Adults Using the ST Genesia Analyzer With the STG‐ThromboScreen Reagent (With and Without Thrombomodulin)
Source: Int J Lab Hematol. 2026 Jun 9;48(5):1189–200. doi: 10.1111/ijlh.70168 (PMC13555124; doi:10.1111/ijlh.70168)
Supplement: Supplementary file 3 [file IJLH-48-1189-s003.docx]

**Supplementary Figure S2.** **Inter-laboratory comparison of daily thrombin generation results.** For each laboratory and plasma pool, three intraday replicate measurements (R1, R2 and R3) were obtained from the same aliquot and averaged to obtain a single daily value. Daily values are displayed for each laboratory. Panel A shows absolute values and Panel B normalized values. Between-laboratory comparisons were performed using the Kruskal–Wallis test followed by Dunn’s post-hoc test with Sidak correction for multiple comparisons.
