# Supplementary material for: Multicenter Interlaboratory Study of Thrombin Generation Assay in Healthy Adults Using the ST Genesia Analyzer With the STG‐ThromboScreen Reagent (With and Without Thrombomodulin)
Source: Int J Lab Hematol. 2026 Jun 9;48(5):1189–200. doi: 10.1111/ijlh.70168 (PMC13555124; doi:10.1111/ijlh.70168)
Supplement: Supplementary file 4 [file IJLH-48-1189-s004.docx]

**Supplementary Table S2.** **Bias analysis.** The relative error for each laboratory, pool and parameter was considered acceptable when the bias was less than 10%.

| **Pool 1** |  |  |  |  |  |  |  |  |  |  |  |  |
| --- | --- | --- | --- | --- | --- | --- | --- | --- | --- | --- | --- | --- |
| **Lab ID** | **Lag Time (min)** | **Peak Height (nM)** | **Time to Peak (min)** | **ETP (nM.min)** | **Vel. Index (nM/min)** | **Start Tail (min)** | **Lag Time (ratio)** | **Peak Height (%)** | **Time to Peak (ratio)** | **ETP (%)** | **Vel. Index (%)** | **Start Tail (ratio)** |
| **1** | 3.08 | 0.16 | 1.46 | 2.07 | -0.79 | 2.62 | 2.95 | -3.97 | 2.02 | -2.21 | -10.46 | 1.75 |
| **2** | -3.99 | -1.05 | -1.81 | -1.87 | -2.01 | -1.75 | -0.86 | -9.20 | 1.44 | -6.30 | -13.42 | 0.79 |
| **3** | 1.55 | -6.45 | 0.64 | -4.38 | -6.41 | 2.65 | 2.09 | 2.92 | 0.43 | 4.39 | 3.51 | 2.60 |
| **4** | 0.39 | 14.76 | -2.15 | 11.91 | 17.42 | -1.72 | -0.28 | 9.60 | -3.42 | 5.38 | 16.65 | -3.10 |
| **5** | 0.58 | -9.01 | 5.94 | -3.70 | -18.70 | 3.36 | -3.26 | 0.78 | 1.83 | 2.20 | -5.03 | -0.21 |
| **6** | 1.55 | 3.54 | -2.78 | 3.39 | 11.46 | 1.05 | -1.25 | 2.39 | -0.55 | 2.20 | 4.80 | 0.80 |
| **7** | 0.02 | -0.47 | 1.25 | 0.48 | -3.26 | -0.09 | -1.58 | -2.17 | 0.50 | -1.05 | -7.07 | 0.33 |
| **8** | -3.91 | 3.42 | 0.23 | 3.52 | -2.93 | -2.22 | -5.26 | 0.48 | -1.49 | -1.10 | -1.45 | -3.48 |
| **9** | 0.63 | 14.04 | -5.21 | 5.25 | 27.65 | -4.65 | 1.87 | 7.74 | -6.16 | -0.90 | 25.80 | -4.62 |
| **10** | -2.79 | -7.49 | -0.45 | -6.41 | -6.03 | 0.81 | 0.32 | -4.72 | 1.17 | 0.00 | -7.00 | 4.47 |
| **11** | 6.66 | -7.10 | 6.65 | -2.95 | -14.66 | 3.54 | 2.63 | 3.80 | 1.73 | 2.18 | 2.99 | -1.63 |
| **12** | -3.76 | -4.35 | -3.77 | -7.30 | -1.73 | -3.61 | 2.63 | -7.64 | 2.49 | -4.80 | -9.32 | 2.30 |
|  |  |  |  |  |  |  |  |  |  |  |  |  |
| **Pool 2** |  |  |  |  |  |  |  |  |  |  |  |  |
| **Lab ID** | **Lag Time (min)** | **Peak Height (nM)** | **Time to Peak (min)** | **ETP (nM.min)** | **Vel. Index (nM/min)** | **Start Tail (min)** | **Lag Time (ratio)** | **Peak Height (%)** | **Time to Peak (ratio)** | **ETP (%)** | **Vel. Index (%)** | **Start Tail (ratio)** |
| **1** | 2.79 | -4.58 | 4.01 | 2.88 | -9.96 | 7.84 | 2.47 | -9.72 | 4.95 | -1.99 | -20.13 | 7.02 |
| **2** | -2.14 | 0.35 | -2.53 | 0.32 | 4.64 | -3.67 | 1.05 | -6.49 | 0.83 | -4.75 | -9.10 | 0.95 |
| **3** | 0.16 | -6.80 | 0.34 | -4.39 | -7.51 | 2.83 | 0.54 | 1.03 | 0.24 | 3.75 | -0.07 | 2.68 |
| **4** | 1.70 | 12.64 | -0.40 | 12.45 | 12.86 | 1.09 | 0.96 | 6.19 | -1.40 | 5.28 | 10.30 | -0.29 |
| **5** | -2.23 | -1.08 | 0.69 | -6.57 | -4.65 | -6.86 | -4.52 | 7.84 | -1.52 | -1.33 | 6.77 | -9.43 |
| **6** | 0.44 | -7.51 | 3.80 | -3.70 | -14.14 | 1.55 | -3.43 | 1.74 | -0.10 | 1.62 | -1.58 | -2.02 |
| **7** | 1.69 | 2.18 | 0.18 | 3.23 | 2.84 | 1.85 | 0.01 | -0.89 | -0.52 | 1.05 | -2.95 | 2.23 |
| **8** | -3.47 | 3.83 | 0.13 | 4.06 | -1.56 | -1.25 | -4.71 | -0.44 | -1.43 | -1.16 | -1.92 | -2.46 |
| **9** | -0.94 | 13.42 | -4.65 | 4.78 | 23.30 | -5.16 | 0.39 | 6.26 | -5.22 | -1.89 | 21.22 | -4.71 |
| **10** | -3.66 | -2.35 | -5.38 | -6.69 | 4.80 | -2.95 | -0.68 | -0.67 | -1.43 | -0.84 | 1.71 | 0.59 |
| **11** | 6.94 | -7.49 | 6.99 | -0.83 | -12.03 | 7.11 | 2.87 | 2.23 | 2.28 | 3.76 | 3.55 | 1.68 |
| **12** | -1.28 | -2.62 | -3.18 | -5.53 | 1.40 | -2.38 | 5.03 | -7.07 | 3.33 | -3.51 | -7.80 | 3.75 |
|  |  |  |  |  |  |  |  |  |  |  |  |  |
| **Pool 3** |  |  |  |  |  |  |  |  |  |  |  |  |
| **Lab ID** | **Lag Time (min)** | **Peak Height (nM)** | **Time to Peak (min)** | **ETP (nM.min)** | **Vel. Index (nM/min)** | **Start Tail (min)** | **Lag Time (ratio)** | **Peak Height (%)** | **Time to Peak (ratio)** | **ETP (%)** | **Vel. Index (%)** | **Start Tail (ratio)** |
| **1** | 3.50 | -3.01 | 2.22 | 1.90 | -4.46 | 5.59 | 3.07 | -7.80 | 3.06 | -2.78 | -14.93 | 4.98 |
| **2** | -3.74 | 7.82 | -4.53 | 1.55 | 12.98 | -5.58 | -0.63 | -1.78 | -1.27 | -3.42 | -1.39 | -2.17 |
| **3** | 0.90 | -11.92 | 1.75 | -6.99 | -12.91 | 5.02 | 1.23 | -4.17 | 1.79 | 0.72 | -5.88 | 4.83 |
| **4** | 0.62 | 15.79 | -0.56 | 16.74 | 15.96 | 1.26 | -0.15 | 9.72 | -1.55 | 9.43 | **13.95** | 0.02 |
| **5** | -0.34 | -5.61 | 1.77 | -6.24 | -9.91 | -2.29 | -2.58 | 3.22 | -0.61 | -0.93 | 0.80 | -4.59 |
| **6** | 1.57 | -9.88 | 3.52 | -5.13 | -14.53 | 3.72 | -2.19 | -0.94 | -0.54 | -0.20 | -1.80 | -0.15 |
| **7** | 1.25 | -1.05 | 0.49 | 1.65 | -1.20 | 2.69 | -0.42 | -3.55 | 0.24 | -0.29 | -6.41 | 3.37 |
| **8** | -3.54 | 7.09 | 0.15 | 6.72 | 1.18 | -2.11 | -4.94 | 3.23 | -1.46 | 1.57 | 1.20 | -3.20 |
| **9** | -2.54 | 11.68 | -2.60 | 5.57 | 18.20 | -4.00 | -1.18 | 5.11 | -3.09 | -1.01 | **16.41** | -3.61 |
| **10** | -1.49 | -0.38 | -5.67 | -6.07 | 9.97 | -4.37 | 1.63 | 1.57 | -1.74 | -0.15 | 6.56 | -0.61 |
| **11** | 5.49 | -9.05 | 7.17 | -4.16 | -18.09 | 4.10 | 1.63 | 0.76 | 2.50 | 0.45 | -3.58 | -0.94 |
| **12** | -1.69 | -1.47 | -3.71 | -5.55 | 2.82 | -4.03 | 4.51 | -5.37 | 2.69 | -3.38 | -4.94 | 2.08 |
