# Supplementary material for: Multicenter Interlaboratory Study of Thrombin Generation Assay in Healthy Adults Using the ST Genesia Analyzer With the STG‐ThromboScreen Reagent (With and Without Thrombomodulin)
Source: Int J Lab Hematol. 2026 Jun 9;48(5):1189–200. doi: 10.1111/ijlh.70168 (PMC13555124; doi:10.1111/ijlh.70168)
Supplement: Supplementary file 5 [file IJLH-48-1189-s005.docx]

**Supplementary Table S1**. **Within-laboratory analysis, expressed by the Precision Reproducibility Index (PRI) of each parameter in all laboratories.** PRI was calculated as the ratio between the CV of a laboratory and the CV of the comparison group (PRI = CVlab / CVgroup). PRI <1 indicates better precision than the comparison group, whereas PRI >1 indicates worse precision. Values between 1 and 2 were considered acceptable, while PRI >2 indicated poor reproducibility and prompted internal review. The table shows how many of the 6 TG parameters evaluated in the study for absolute and normalized values presented a PRI <1, between 1-2 or >2.

|  |  | **Absolute values** | | | **Normalizad values** | | |
| --- | --- | --- | --- | --- | --- | --- | --- |
| Laboratory | Pool | PRI <1 | PRI 1-2 | PRI >2 | PRI <1 | PRI 1-2 | PRI >2 |
| 1 | pool 1 | 5/6 | 1/6 | 0/6 | 5/6 | 1/6 | 0/6 |
|  | pool 2 | 6/6 | 0/6 | 0/6 | 5/6 | 1/6 | 0/6 |
|  | pool 3 | 5/6 | 1/6 | 0/6 | 6/6 | 0/6 | 0/6 |
| 2 | pool 1 | 6/6 | 0/6 | 0/6 | 5/6 | 1/6 | 0/6 |
|  | pool 2 | 6/6 | 0/6 | 0/6 | 6/6 | 0/6 | 0/6 |
|  | pool 3 | 4/6 | 2/6 | 0/6 | 1/6 | 3/6 | 2/6 |
| 3 | pool 1 | 1/6 | 4/6 | 1/6 | 0/6 | 4/6 | 2/6 |
|  | pool 2 | 5/6 | 1/6 | 0/6 | 5/6 | 1/6 | 0/6 |
|  | pool 3 | 6/6 | 0/6 | 0/6 | 0/6 | 6/6 | 0/6 |
| 4 | pool 1 | 4/6 | 2/6 | 0/6 | 5/6 | 1/6 | 0/6 |
|  | pool 2 | 5/6 | 1/6 | 0/6 | 1/6 | 1/6 | 0/6 |
|  | pool 3 | 2/6 | 4/6 | 0/6 | 2/6 | 2/6 | 1/6 |
| 5 | pool 1 | 6/6 | 0/6 | 0/6 | 5/6 | 1/6 | 0/6 |
|  | pool 2 | 3/6 | 3/6 | 0/6 | 1/6 | 5/6 | 0/6 |
|  | pool 3 | 3/6 | 3/6 | 0/6 | 1/6 | 5/6 | 0/6 |
| 6 | pool 1 | 3/6 | 2/6 | 1/6 | 2/6 | 4/6 | 0/6 |
|  | pool 2 | 5/6 | 1/6 | 0/6 | 6/6 | 0/6 | 0/6 |
|  | pool 3 | 5/6 | 1/6 | 0/6 | 5/6 | 1/6 | 0/6 |
| 7 | pool 1 | 5/6 | 1/6 | 0/6 | 5/6 | 1/6 | 0/6 |
|  | pool 2 | 6/6 | 0/6 | 0/6 | 6/6 | 0/6 | 0/6 |
|  | pool 3 | 6/6 | 0/6 | 0/6 | 4/6 | 2/6 | 0/6 |
| 8 | pool 1 | 6/6 | 0/6 | 0/6 | 5/6 | 1/6 | 0/6 |
|  | pool 2 | 6/6 | 0/6 | 0/6 | 6/6 | 0/6 | 0/6 |
|  | pool 3 | 6/6 | 0/6 | 0/6 | 0/6 | 0/6 | 0/6 |
| 9 | pool 1 | 3/6 | 1/6 | 2/6 | 5/6 | 1/6 | 0/6 |
|  | pool 2 | 2/6 | 4/6 | 0/6 | 1/6 | 4/6 | 1/6 |
|  | pool 3 | 3/6 | 3/6 | 0/6 | 1/6 | 3/6 | 2/6 |
| 10 | pool 1 | 4/6 | 2/6 | 0/6 | 4/6 | 1/6 | 1/6 |
|  | pool 2 | 6/6 | 0/6 | 0/6 | 2/6 | 3/6 | 1/6 |
|  | pool 3 | 5/6 | 1/6 | 0/6 | 4/6 | 1/6 | 1/6 |
| 11 | pool 1 | 3/6 | 2/6 | 1/6 | 1/6 | 5/6 | 0/6 |
|  | pool 2 | 5/6 | 0/6 | 1/6 | 3/6 | 2/6 | 0/6 |
|  | pool 3 | 3/6 | 2/6 | 1/6 | 0/6 | 0/6 | 0/6 |
| 12 | pool 1 | 5/6 | 1/6 | 0/6 | 4/6 | 2/6 | 0/6 |
|  | pool 2 | 6/6 | 0/6 | 0/6 | 2/6 | 3/6 | 1/6 |
|  | pool 3 | 6/6 | 0/6 | 0/6 | 1/6 | 4/6 | 0/6 |
